# Can non-fatal burden estimates from the Global Burden of Disease study be used locally? An investigation using models of stroke and diabetes for South Africa

**DOI:** 10.1080/16549716.2020.1856471

**Published:** 2021-01-04

**Authors:** Victoria Pillay-van Wyk, Rifqah Abeeda Roomaney, Mweete Debra Nglazi, Oluwatoyin Folashade Awotiwon, Judith M Katzenellenbogen, Tracy Glass, Janetta Debora Joubert, Debbie Bradshaw

**Affiliations:** a Burden of Disease Research Unit, South African Medical Research Council, Cape Town, South Africa; b School of Population and Global Health, The University of Western Australia, Australia

**Keywords:** Prevalence-based years of life lived with disability, stroke, diabetes, disease modelling, non-fatal burden

## Abstract

**Background**: The Global Burden of Disease (GBD) approach estimates disease burden by combining fatal (years of life lost) and non-fatal burden prevalence-based years of life lived with disability (PYLDs) estimates. Although South Africa has data to estimate mortality, prevalence data to estimate non-fatal burden are sparse. PYLD estimates from the GBD study for South Africa can potentially be used. However, there is a divergence in mortality estimates for South Africa between the second South African National Burden of Disease (SANBD2) and 2013 GBD studies.

**Objective**: We investigated the feasibility of utilising GBD PYLD estimates for stroke and diabetes by exploring different disease modelling scenarios.

**Method**: DisMod II software-generated South African stroke and diabetes PYLDs for 2010 from models using local epidemiological parameters and demographic data for people 20–79 years old. We investigated the impact on PYLD estimates of 1) differences in the cause-of-death envelope, 2) differences in the cause-specific mortality estimates (increase/decrease by 15% for stroke and 30% for diabetes), and 3) difference using local disease parameters compared to other country or region parameters. Differences were expressed as ratios, average ratios and ratio ranges.

**Results**: Using the GBD cause-of-death envelope (16% more deaths than SANBD2) and holding other parameters constant yielded age-specific ratios of PYLDs for stroke and diabetes ranging between 0.89 and 1.07 (average 0.98) for males. Similar results were observed for females.

A 15% change in age-specific stroke mortality showed little difference in the ratio comparison of PYLDs (range 0.98–1.02) while a 30% change in age-specific diabetes mortality resulted in a ratio range of 0.96–1.07 for PYLDs depending on age.

**Conclusion**: This study showed that GBD non-fatal burden estimates (PYLDs) can be used for stroke and diabetes non-fatal burden in the SANBD2 study.

## Background

South Africa is an upper middle-income country facing a quadruple mortality burden due to continued high levels of i) HIV/AIDS and tuberculosis; ii) other communicable diseases, perinatal conditions, maternal causes, and nutritional deficiencies; iii) non-communicable diseases; and iv) injuries [1]. The poor health status of the country is in part due to the history of colonialism and apartheid (where every aspect of life was racially segregated), through the exploitation of the working class, differential access to health services and extreme wealth inequalities. High levels of poverty, unemployment and unequal access to health services persist [2–4]. Knowledge and understanding of changes in the country’s disease burden will enable national and provincial governments to apply evidence-based health policies and resource allocation rather than using historical budgeting approaches or merely reacting to demands placed upon the health system. Together with information about the cost-effectiveness of interventions, estimates of the disease burden can be utilised to set priorities to improve health.

The Institute of Health Metrics and Evaluation’s Global Burden of Disease (GBD) study is a comprehensive worldwide observational epidemiological study that quantifies the total burden of disease experienced in countries by combining mortality (years of life lost, YLLs) and non-fatal burden (prevalent years lived with a disability, PYLDs) from major diseases, injuries and risk factors at global, regional and national levels, including South Africa. Given that health data are sparse in many parts of the world, a specialised disease modelling software tool, DisMod II, was developed for national burden of disease (NBD) studies using a set of mathematical equations to calculate the complete epidemiology of a disease including the incidence, prevalence and mortality for a given location [5,6]. To do this, DisMod II requires at least three disease-specific epidemiological values as inputs, as well as all-cause mortality rates and the population structure pertaining to that location [6]. The second South African National Burden of Disease (SANBD2) study is currently quantifying the national non-fatal burden, using burden of disease methodology. This study has already quantified the mortality burden for South Africa [1] using vital registration data from Statistics South Africa (Stats SA), however, data to estimate non-fatal burden are limited for the country [7]. It is therefore important to assess how the existing extensive collation and review of global non-fatal epidemiological data for all diseases undertaken by the GBD study can be harnessed for South Africa.

Previous comparison between South African mortality estimates from the SANBD2 [1] and GBD 2013 studies for the year 2010 revealed differences in the overall mortality estimates and cause of death profiles for South Africa. The GBD 2013 study estimated 96,000 more deaths compared to the SANBD2 study and therefore used a different cause of death envelope for 2010.^1^^1^Email communication Prof Haidong Wang, Demographer, Institute of Health Metrics and Evaluation, University of Washington, 2014. This is mainly due to the divergence between the estimates of HIV/AIDS deaths that has had a significant impact on the South African burden of disease estimates. Both GBD 2013 and the SANBD2 studies rank HIV/AIDS as the leading causes of death, however GBD 2013 reported over 65 000 more deaths than SANBD2 for HIV/AIDS. This overestimation of HIV/AIDS deaths for South Africa in GBD 2013 [1,8] can be attributed to the use of the UNAIDS Spectrum model [9] which is known to have a limited ability to evaluate the impact of HIV prevention strategies [10,11]. This has presented challenges for the GBD study in estimating HIV/AIDS deaths for South Africa.

Furthermore, the SANBD2 study shows HIV/AIDS deaths peaked in 2005/6 [12] followed by a rapid decrease which is in line with the rollout of antiretroviral treatment in the country while the GBD 2013 study shows HIV/AIDS deaths peaked in 2011/2012 [8]. Since 29% of deaths in South Africa were from HIV/AIDS [1], these deaths have a marked impact on the overall cause of death envelope for the country, and consequences for the magnitude and ranking of other single causes of death. This is noted for stroke and diabetes, common health problems in South Africa that ranked differently and were of different magnitude in the two studies for 2010.

Moreover, the GBD statistical modelling approach fills data gaps for countries and regions with no or limited available data by using the data from the closest region to compensate for lack of data. Therefore, some of the country and regional estimates are generated from non-local data, which can result in inappropriate estimates when the source data do not reflect the epidemiology of the disease in the index country/region.

Besides disease-specific input parameters (for example, incidence, prevalence), DisMod II also draws on population-specific demographic information, including all-cause mortality rates and the age and sex distribution in the population. Thus, it can be expected that use of different mortality profiles in DisMod II will influence the epidemiological outputs produced, which in turn will impact the PYLD estimates.

Given the dearth of non-fatal data in South Africa, it would be useful to assess whether GBD mortality estimates can be used for estimating PYLDs despite the differences in the mortality between GBD and SA studies. This paper thus aims to investigate whether or not different cause of death envelopes and/or different cause-specific mortality estimates produce comparable PYLD estimates using the examples of stroke and diabetes. The study will also investigate the impact of using a non-South African relative risk mortality (a disease-specific input to DisMod II that indicates the excess mortality from that disease) to generate local PYLDs for stroke and diabetes. The disease models for these conditions have previously been published by Bertram et al. [13,14] and will be modified in this analysis.

## Methods

The published stroke and diabetes models by Bertram et al. [13,14] used available South African data inputs and non-local data; these models will be used as the basis for this investigation. Bertram et al. [13,14] reported incident YLDs, whereas this paper reports on PYLDs.

### Published models

Both the published stroke and diabetes models used the AIDS and Demographic model of the Actuarial Society of South Africa (ASSA2008) [15] as the source of data for population size and age-sex distribution of the 2009 population. These models also used age-specific all-cause mortality rates for 2009 from the SANBD2 study.

Disease-specific input parameters used for the published stroke model were: i) prevalence from the South African Demographic Household Survey 2003 [16], ii) relative risk mortality (i.e. mortality from the disease (stroke) population relative to the total population) from the Western Australian Burden of Disease study [17,18] and iii) zero remission (Table 1). Disease-specific input parameters for the published diabetes model were: i) pooled prevalence for diabetes from various local studies [19–22], ii) relative risk mortality from Asia Pacific Cohort Studies Collaboration [23] and iii) zero remission (Table 1).

**Table 1.** Summarized models

			DisMod II INPUT PARAMETERS							
	ENVELOPE		Prevalence		Relative risk mortality		Age-specific cause-specific mortality rate		Remission	
MODEL	Population structure	All-cause mortality rate	Stroke	Diabetes	Stroke	Diabetes	Stroke	Diabetes	Stroke	Diabetes
1	Alternative mid-year population estimates [24]	2010 from NBD 2010 study	Bertram and Katzenellenbogen et al 2013 [13]	Bertram and Jawal et al 2013 [14]	Asia Western Australian BOD study [17]	Asia Pacific Cohort Studies Collaboration [23]			Zero	Zero
2	2010 from GBD 2013 study	2010 from GBD 2013 study								
3	Alternative mid-year population estimates [24]	2010 from NBD 2010 study					2010 from SANBD2 study	2010 from SANBD2 study		
4							30% increase in cause-specific mortality rate for each age group	15% increase in cause-specific mortality rate for each age group		
5							30% decrease in cause-specific mortality rate for each age group	15% decrease in cause-specific mortality rate for each age group		

In DisMod II remission is defined as ‘cured’, hence remission is assumed to be zero when modelling chronic conditions like stroke and diabetes.

### Comparison using varying input parameters

Different input parameters were used in five separate models, to investigate the impact on estimates of PYLDs of i) a different cause of death envelope, ii) different cause-specific mortality, and iii) input parameters from South Africa versus other country/region parameters. All models were generated for people 20 –79 years of age by sex. These models are discussed below and a summary of the inputs into the different models is reported in Table 1.

### i. Investigation of difference in cause of death envelopes: SANBD2 vs GBD 2013

For Model 1, mid-year population estimates prepared by Dorrington[24] and age-specific all-cause mortality from the SANBD2 study [1] were used as population-specific demographic inputs for 2010 in DisMod II. This was compared with Model 2, which used population-specific demographic information for 2010 estimated for South Africa by the GBD 2013 study.^2^^2^Email communication Prof Haidong Wang, Institute of Health Metrics and Evaluation, University of Washington, 2014. These GBD death counts and population estimates had been used to calculate the GBD age-specific all-cause mortality rate for South Africa.

### ii. Investigation of differences in cause-specific mortality

Models 3–5 were developed to measure the impact of different age- and cause-specific mortality rates on estimates of PYLDs for stroke and diabetes.

Model 3 was developed to estimate PYLDs for 2010 using only local disease-specific input parameters in DisMod II and using the same population specific demographic information as Model 1

Since no additional data were found for incidence, prevalence or relative risk mortality for South Africa for stroke or diabetes, the same prevalence and remission from Model 1 were used as disease-specific input parameters in DisMod II. However, for both stroke and diabetes, the foreign relative risk mortality was replaced by the South African age-specific cause-specific mortality rate from the SANBD2 study.

Models 4 and 5 used the same population-specific demographic information and disease-specific input parameters in DisMod II as Model 3 for stroke and diabetes, except that these models built in some level of change in cause-specific mortality rate. In particular, Model 4 was developed to measure the impact of a 15% and 30% increase in the age-specific mortality rates for stroke and diabetes, respectively, while Model 5 measured the impact of 15% and 30% decrease in the age-specific stroke and diabetes mortality rates respectively.

### iii. Investigation of differences using local parameters versus other country or region parameters

Models 1 and 3 were compared to investigate the impact of using local parameters versus other country or region parameters on estimates of PYLDs.

The variation between Models was calculated as ratio comparisons using the formula below:

$$Ratio=PYLDs_{Model\;X}/PYLDs_{Model\;Y};$$

*where X and Y depict the different scenario models.*

Ratio averages were calculated to summarise ratios across age groups using the following formula:

*where* N *depicts the number of age groups.*

Ratio ranges were used to capture the highest and lowest ratios across the age groups. Age-specific PYLD rates were calculated using the mid-year population estimates by Dorrington [24].

### Results

Table 2 reports the ratio comparison of the PLYDs for stroke and diabetes for the different scenarios modelled. Using the GBD 2013 study cause-of-death envelope (16% more deaths than SANBD2 study) and holding other parameters constant (Model 1 versus Model 2), yielded ratios of PYLDs for stroke and diabetes ranging between 0.89 and 1.07 (ratio average 0.98) for males (Table 2). Similar results were observed for females.

**Table 2.** Ratio comparison of PYLD models for stroke and diabetes for 20 –79 year olds

	Stroke				Diabetes			
	Males		Females		Males		Females	
Model comparison	Ratio Average	Ratio Range	Ratio Average	Ratio Range	Ratio Average	Ratio Range	Ratio Average	Ratio Range
Different cause-of-death envelope *Model 1 versus Model 2*	0.98	0.89–1.07	0.98	0.89–1.06	0.98	0.89–1.07	0.98	0.89–1.06
Local parameters and other country parameters *Model 1 versus Model 3*	1.07	0.94–1.59	1.02	0.92–1.29	1.03	0.92–1.20	0.98	0.94–1.30
Increased age-specific mortality rate *Model 3 versus Model 4*	1.0	0.99–1.02	1.0	1.0–1.0	1.01	0.98–1.07	1.01	1.0–1.05
Decreased age-specific mortality rate *Model 3 versus Model 5*	1.0	0.98–1.01	1.0	1.0–1.0	1.0	0.96–1.07	0.99	0.96–1.0

A 15% increase (Models 3 vs 4; ratio range 0.99-1.02) or decrease (Models 3 vs 5; ratio range: 0.98-1.01) in age-specific stroke mortality rate showed no difference in the ratio comparison of PYLDs for males (ratio average 1.0) when compared to the SANBD2 2010 age-specific stroke mortality rate; similar findings were observed for females. Very slight differences were observed for diabetes PYLDs among males and females when comparing a 30% increase or 30% decrease in age-specific diabetes mortality rate with the SANBD2 2010 age-specific diabetes mortality rate (Table 2).

**Figure 1.**
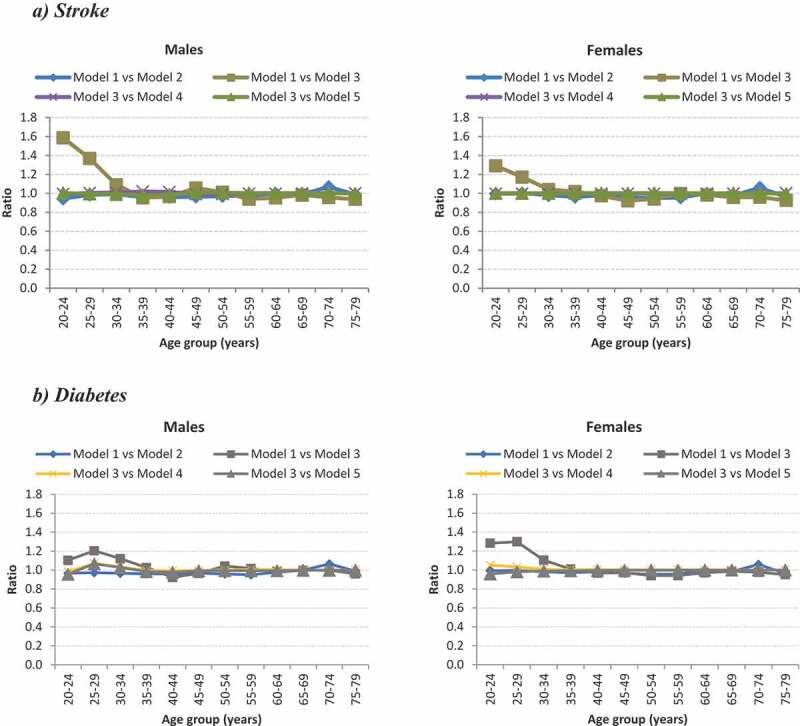
Ratio comparison of PYLD models for ***stroke*** and diabetes by age and sex for 2010

Note: Model 1 vs Model 2: Differences in cause of death envelope; Model 1 vs Model 3: Local parameters and other country parameters; Model 3 vs Model 4:Increased age-specific cause mortality rate; Model 3 vs Model 5: Decreased age-specific cause mortality rate

The use of a foreign relative risk mortality (i.e. Western Australia for stroke and Asia Pacific for diabetes) compared to using only local parameters to estimate PYLDs revealed some differences in estimates in the younger ages for both stroke and diabetes (Figure 1). For both the stroke and diabetes models, using only local data as input parameters (Model 3) generated fewer PYLDs in the younger ages when compared to using non-local data (Model 1). Minor differences were observed in the older ages (>30 years) for all models.

**Figure 2.**
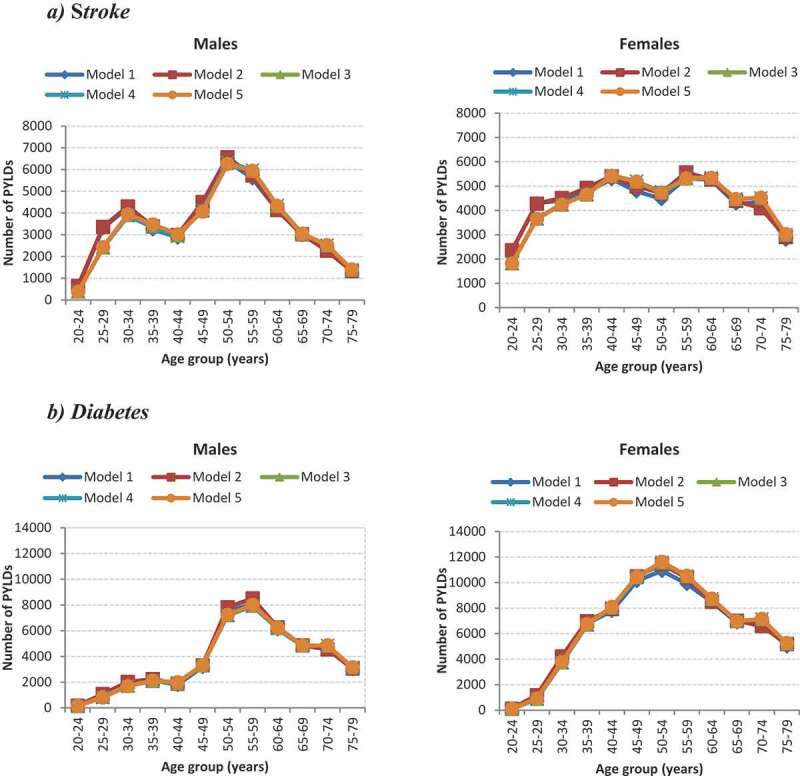
Estimated PYLDs by age and sex from different models for ***stroke*** and diabetes

Figure 2 displays the estimated PYLDs from the different models for stroke and diabetes by age and sex. All models for stroke and diabetes by age and sex generated similar numbers of PYLDs.

**Figure 3.**
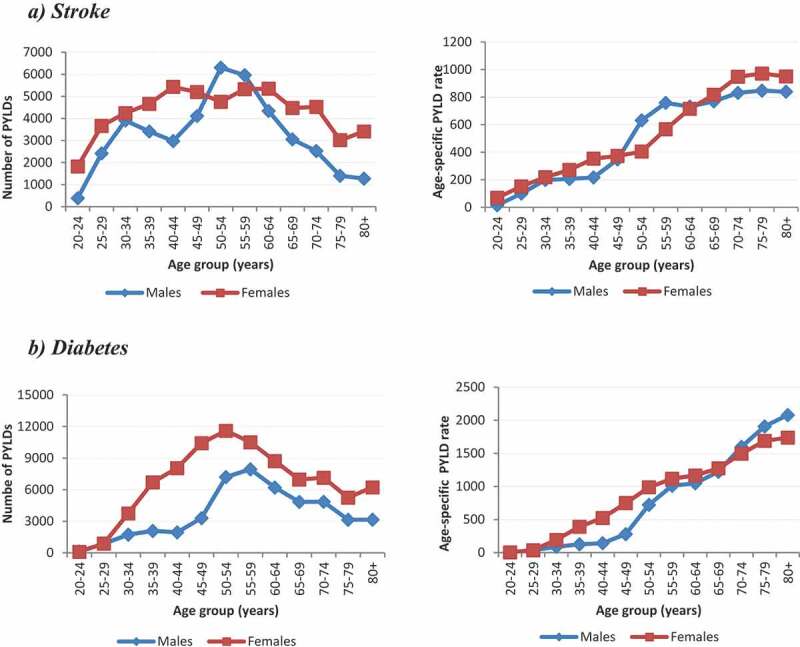
The number of PYLDs and age-specific cause-specific rates for the ***stroke*** (a) and diabetes (b): South Africa 2010 (Model 3)

Model 3 uses only local parameters for the estimation of PYLDs and thus reflects PYLDs for South Africa. The number of PYLDs and the age-specific PYLD rate for the stroke and diabetes models for 2010 are displayed in Figure 3 (Model 3). For stroke, females had higher PYLD counts in all ages, except for 50 –59 year olds, when compared to males. Despite the bimodal peaks in counts at 30 –34 years and 55 –59 years in males, a steady increase in the age-specific PYLD rates was observed for both males and females.

For diabetes, the number of PYLDs peak at 50 –54 years for females and 55 –59 years for males. Females had higher numbers of PYLDs than males across all ages and females younger than 69 years of age had higher age-specific PYLD rates than males.

### Discussion

Our study showed that using different cause of death envelopes and cause-specific mortality estimates did not result in substantially different PYLD estimates. This key finding indicates that the differences in mortality profile [1,8], highlighted in the background of this paper, between the GBD 2013 study and SANBD2 study does not have a substantial impact on the non-fatal burden estimates. Clearly, the incidence and prevalence of conditions are the strong determinants of the PYLD. This implies that the GBD non-fatal estimates can be used in the SANBD2 study [25].

Our investigation into the impact of non-South African disease-specific input parameters on the estimation of PYLDs showed differences in PLYDs in the younger ages in both the stroke and diabetes models; albeit when PYLD rates are lowest. This indicates that the Asia Pacific Cohort Collaboration [23] and Western Australia [17,18] relative risk mortality is not consistent with the local cause-specific mortality rates in the younger ages. It is not clear what factors are related to this difference. Local epidemiological studies would provide valuable information on excess mortality that would better inform such disease modelling.

Studies of the non-fatal burden of disease in the South African setting are constrained by the limited availability of data on the incidence and prevalence of conditions [7,26], as well as disease outcomes. Our investigation built on existing work, using two of the few disease models developed in the South African setting using the burden of disease approach. This study has applied a systematic evaluation of the epidemiological estimation of PYLDs using disease parameters and specific demographic information for 2010; even though data are not contemporary, the purpose of this work was a methodological investigation into disease modelling to assess the feasibility and utility of using GBD estimates in conjunction with country-specific data to obtain robust estimates of disease burden. Such methodological issues *are* independent of the time and remain relevant particularly in countries where demographic and non-fatal health data are sparse. DisMod-MR, a Bayesian meta-regression, version of the disease model has been developed for subsequent global studies [27]. Designed to pool data from different countries and provide uncertainty estimates, the tool requires significant computing power and a desktop version is currently not available. True cohort effects are not included in either DisMod II nor DisMod-MR, limiting their use for estimation of diseases with rapidly changing incidence rates e.g. HIV and require a full age-time-period model. Nonetheless, DisMod II is adequate for a single country setting and has enabled us to evaluate the impact of observed differences in mortality estimates for South Africa on the PYLDs.

In conclusion, this study showed that GBD non-fatal burden estimates (PYLDs) can be used for stroke and diabetes non-fatal burden in the SANBD2 study. Like South Africa, many countries have limited epidemiological data for estimating non-fatal burden. Where possible, these countries conducting National Burden of Disease studies, using local data inputs for mortality burden, should assess the feasibility of using GBD estimates for their non-fatal burden.

## Glossary

**DisMod II**

A software tool that can be used to check the consistency of the epidemiological parameters (incidence, prevalence, duration and case fatality) of a disease based on a multi-state life table model of a single disease, together with mortality from all other causes for a specified population. The population cohort components include individuals who are susceptible, affected, have died or moved to a state of remission.

**Global burden of disease (GBD)**

A comprehensive demographic and epidemiological framework to estimate health gaps for an extensive set of disease and injury causes, and for major risk factors, using all available mortality and health data and standardised methods to ensure internal consistency and comparability of estimates.

**National burden of disease study (NBD)**

A comprehensive demographic and epidemiological framework to estimate health gaps for a country for an extensive set of disease and injury causes, and for major risk factors, using all available mortality and health data and methods to ensure internal consistency and comparability of estimates.

**Prevalent Years lived with disability (PYLD)**

The loss of healthy life through non-fatal health conditions, calculated from the prevalence and severity of the condition.

**Spectrum Model**

SPECTRUM is a suite of demographic and disease models originally developed to assess the impact of HIV and AIDS and guide policy. The suite now includes many other health and population policy-relevant tools.

**Years of life lost (YLL)**

The years of life lost, compared to a normative standard, due to premature mortality. This is a measure of the mortality gap.

## Paper context

South Africa has limited non-fatal data to generate burden of disease estimates. Non-fatal data from the GBD studies could potentially be used for burden of disease estimation for South Africa. Previous work showed differences between mortality estimates from the SANBD2 and GBD studies. We investigated the feasibility of utilising GBD PYLDs for diabetes and stroke by exploring different disease modelling scenarios and found that GBD stroke and diabetes PYLDs can be used in the SANBD2 study.
